# Basal forebrain volume and metabolism in carriers of the Colombian mutation for autosomal dominant Alzheimer’s disease

**DOI:** 10.1038/s41598-024-60799-9

**Published:** 2024-05-17

**Authors:** Stefan Teipel, Alice Grazia, Martin Dyrba, Michel J. Grothe, Nunzio Pomara

**Affiliations:** 1 Deutsches Zentrum für Neurodegenerative Erkrankungen (DZNE), Gehlsheimer Str. 20, 18147 Rostock, Germany; 2 Department of Psychosomatic Medicine, University Medicine Rostock, Gehlsheimer Str. 20, 18147 Rostock, Germany; 3 CIEN Foundation/Queen Sofia Foundation Alzheimer Center, Madrid, Spain; 4 Geriatric Psychiatry Division, Nathan Kline Institute/Department of Psychiatry and Pathology, NYU Grossman School of Medicine, Orangeburg, NY USA

**Keywords:** Cognitive ageing, Genetics of the nervous system, Dementia, Neurodegenerative diseases

## Abstract

We aimed to study atrophy and glucose metabolism of the cholinergic basal forebrain in non-demented mutation carriers for autosomal dominant Alzheimer's disease (ADAD). We determined the level of evidence for or against atrophy and impaired metabolism of the basal forebrain in 167 non-demented carriers of the Colombian PSEN1 E280A mutation and 75 age- and sex-matched non-mutation carriers of the same kindred using a Bayesian analysis framework. We analyzed baseline MRI, amyloid PET, and FDG-PET scans of the Alzheimer’s Prevention Initiative ADAD Colombia Trial. We found moderate evidence against an association of carrier status with basal forebrain volume (Bayes factor (BF_10_) = 0.182). We found moderate evidence against a difference of basal forebrain metabolism (BF_10_ = 0.167). There was only inconclusive evidence for an association between basal forebrain volume and delayed memory and attention (BF_10_ = 0.884 and 0.184, respectively), and between basal forebrain volume and global amyloid load (BF_10_ = 2.1). Our results distinguish PSEN1 E280A mutation carriers from sporadic AD cases in which cholinergic involvement of the basal forebrain is already detectable in the preclinical and prodromal stages. This indicates an important difference between ADAD and sporadic AD in terms of pathogenesis and potential treatment targets.

## Introduction

Pathological evidence suggests a cholinergic deficit in dementia stages of sporadic Alzheimer’s disease (AD), characterized by reduced choline-acetyl transferase and acetylcholinesterase activity in cortical target regions of cholinergic projections from the basal forebrain, particularly the Nucleus basalis Meynert (NbM), and loss of cholinergic neurons in the NbM^1^. In early stages of sporadic AD, i.e. in people with an antemortem diagnosis of mild cognitive impairment (MCI), cholinergic basal forebrain neurons exhibited neurofibrillary tangles and cell shrinkage associated with accumulation of cortical amyloid^2,3^, but not yet frank neuron loss^4^.

Consistently, volumetric MRI studies demonstrated atrophy of the basal forebrain in sporadic MCI cases^5–9^ and amyloid positive cognitively normal people^10–12^. FDG-PET studies found increased basal forebrain metabolic rate in sporadic MCI cases compared to normal controls^13,14^, which may indicate compensatory upregulation of regional metabolism in early stages of neurodegeneration or loss of cortical GABAergic inhibitory neurons. In contrast to sporadic AD, basal forebrain has not yet been studied in humans with autosomal dominant AD (ADAD).

Here, we studied baseline data from participants of the API Colombian trial recruited from a kindred harboring the Colombian NM_000021:c.839A>C, p.(Glu280Ala) (commonly known as PSEN1 E280A) mutation^15^, which is associated with early onset ADAD^16^. We determined volume and metabolism of the cholinergic basal forebrain in association with mutation carrier status and levels of cerebral amyloid. We hypothesized that non-demented PSEN1 E280A mutation carriers would exhibit atrophy and increased glucose metabolism of the basal forebrain compared with non-carriers, suggesting an early cholinergic deficit and compensatory hyperactivity. For comparison, we examined hippocampal atrophy, as some^17,18^, but not all^19,20^ studies have shown hippocampal atrophy in prodromal ADAD. In addition, we used thalamus as comparison region that had been found to be atrophied in different presenilin mutations^21–24^. We used a Bayesian analysis framework for two reasons. First, a Bayesian approach allowed us to directly quantify evidence for and against an effect. Thus, in case of absence of an effect, we could directly quantify how plausible the evidence was for the absence of an effect^25,26^. This is different for the frequentist null hypothesis significance testing. Here, the p value indicates the probability with which the same or an even more extreme effect will be found in hypothetical repetitions of the same experiment if the hypothesis of no effect is true^27^. Secondly, the Bayesian credible interval represents the bounds within which the true value is expected to lie with 95% probability given the observed data (^28^, chapter 11.3). The frequentist confidence interval relates to long-term realizations of the parameter value in future hypothetical experiments^29^. Given this rather non-intuitive meaning, the frequentist confidence interval is often misinterpreted as if it were a Bayesian credible interval. Evidence for early cholinergic changes in autosomal dominant AD would underscore the phenotypic similarity between sporadic AD and ADAD, whereas evidence for the absence of such effects would point to important differences between ADAD and sporadic AD in terms of pathogenesis and treatment targets^30^.

## Results

### Demographics

Overall 242 cases, including 167 mutation carriers and 75 non-mutation carrier family members, met inclusion criteria. Evidence was extremely in favor of a higher age in the non-carriers, but evidence was inconclusive or absent for a difference in sex, years of education, MMSE, and CDR scores (see Table 1 for details).

**Table 1:** Demographic characteristics.

	PSEN1 E280A carriers	Noncarriers
N (female/male)^1^	101/66	50/25
Age group (30–34/35–39/40–44/45–49/50–54 years)^2^	71/48/30/17/1	9/18/19/20/9
CDR global (0/0.5)^3^	150/17	70/5
MMSE score (95% credible interval)^4^	28.8 (28.6–29.0)	29.2 (29.0–29.4)
Education (95% credible interval) [years]^5^	8.8 (8.1–9.4)	8.5 (7.5–9.5)

^1^Bayesian contingency table: Bayes factor shows moderate evidence for no difference in proportion of sex between groups (BF_10_ = 0.251).

^2^Bayesian contingency table: Bayes factor shows extreme evidence for a difference in proportion of age-groups between groups (BF_10_ = 3.8 × 10^6^), with higher age in the noncarriers.

^3^Bayesian contingency table: Bayes factor shows moderate evidence for no difference in proportion of CDR global scores between groups (BF_10_ = 0.138).

^4^Bayesian *t*-test: Bayes factor shows inconclusive evidence for a difference between groups (BF_10_ = 1.384).

^5^Bayesian *t*-test: Bayes factor shows moderate evidence for no difference between groups (BF_10_ = 0.168).

### Association of carrier status with brain volumes

**Figure 1:**
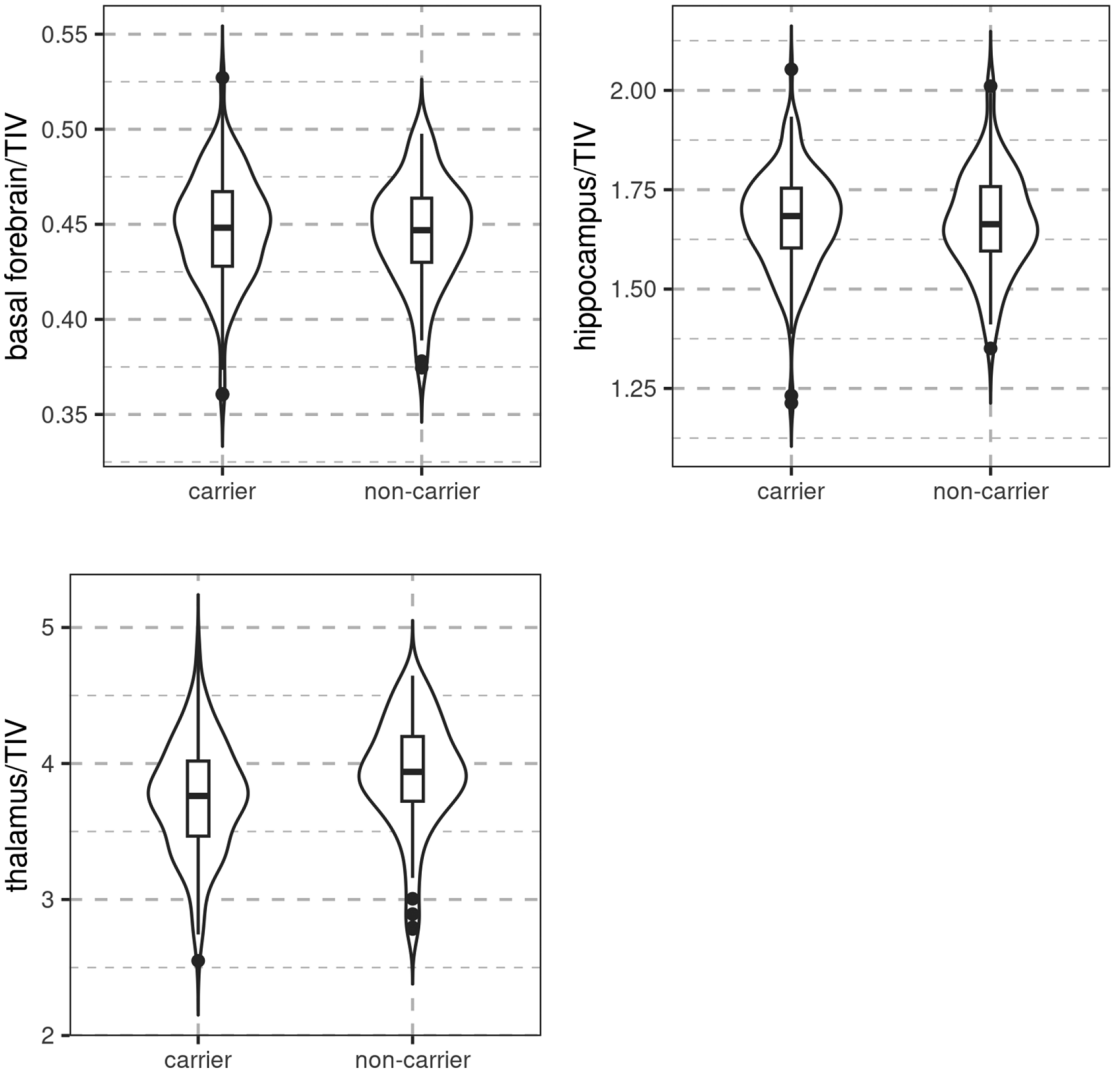
Association of carrier status with brain volumes. Boxplots and violin plots featuring volumes of basal forebrain, hippocampus, and thalamus according to mutation carrier status. Each volumetric measure was normalized to total intracranial volume (TIV).

We found moderate evidence for no association of carrier status with normalized basal forebrain volume (BF_10_ = 0.182) or hippocampus volume (BF_10_ = 0.163), controlling for age, sex, and CDR score. When we repeated this analysis with an unidirectional prior derived from our a priori hypothesis, i.e. carriers were expected to have smaller volumes than non-carriers, the evidence for absence of an effect became even stronger with a BF_10_ of 0.104 for basal forebrain, and a BF_10_ of 0.116 for hippocampus, i.e. absence of a smaller basal forebrain or hippocampus volume in carriers than in non-carriers was 8.6 to 9.6 times more likely than presence of such an effect. We found moderate evidence for an smaller thalamus volume in mutation carriers (BF_10_ = 8.38), controlling for age group, sex, and CDR score. This effect was preserved with a BF_10_ of 7.8 after including ApoE epsilon 4 genotype into the null model. Details are shown in Fig. 1.

### Association of carrier status and brain volumes with cognitive scores

**Figure 2:**
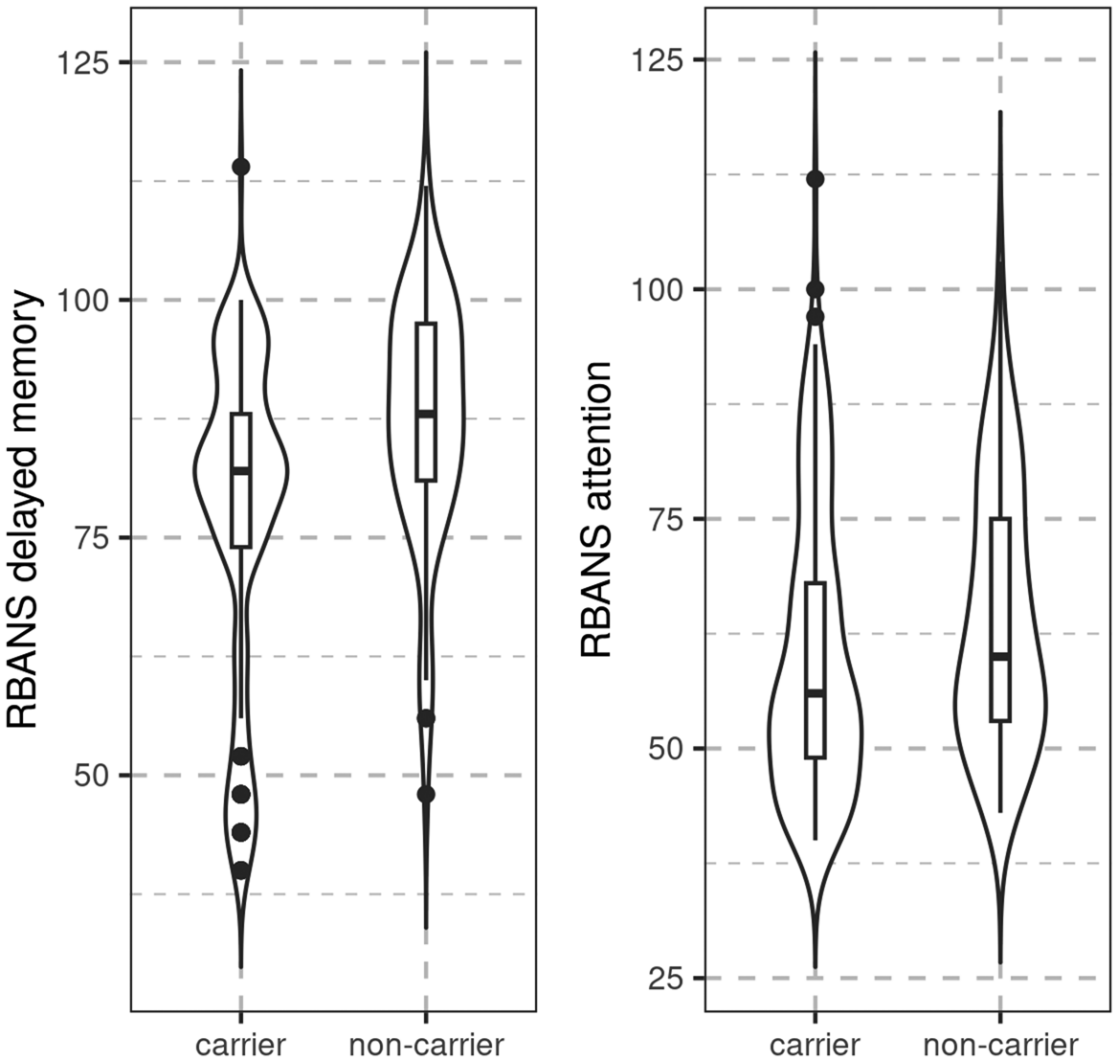
Association of carrier status with cognitive scores. Boxplots and violin plots featuring distribution of cognitive scores of delayed recall memory and attention according to mutation carrier status.

Within the CDR 0 cases, we found extreme evidence for an association of carrier status with delayed recall performance (RBANS delayed memory) (BF_10_ = 12,062.2), indicating poorer performance in mutation carriers, controlling for age group, education, and sex. Evidence was inconclusive for attention (BF_10_ = 0.454). Details are shown in Fig. 2.

**Figure 3:**
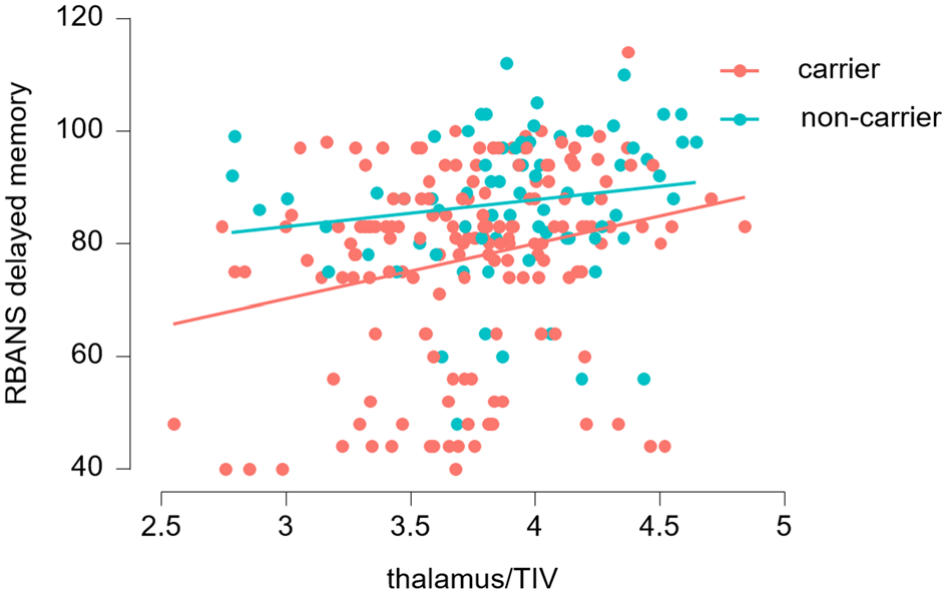
Association of thalamus volume with delayed recall across. Scatter plot of delayed recall regressed on thalamus volume (normalized to total intracranial volume) split according to mutation carrier status.

Both in CDR 0 and in all cases combined, we found evidence against an association of normalized basal forebrain and hippocampus volumes with delayed recall. Evidence was in favor of an association of thalamus volume with delayed recall (Fig. 3), both in CDR 0 and in all cases combined, but not with attention, after controlling for age, sex, education, and carrier status. Details are shown in Table 2. Numbers in the CDR 0.5 subgroup were too small to run meaningful regression analyses separately in this group.

**Table 2:** Association of brain volumes with cognitive scores.

	Delayed recall β [95% credible interval] + BF_10_	Attention β [95% credible interval] + BF_10_
CDR 0 cases		
Basal forebrain	53.9 [− 14.7 to 122.5] BF_10_ = 0.686	− 50.1 [− 112.0 to 11.5] BF_10_ = 0.508
Hippocampus	13.6 [− 2.2 to 29.4] BF_10_ = 0.996	− 7.4 [− 21.3 to 6.5] BF_10_ = 0.246
Thalamus	6.2 [1.6 to 10.8] BF_10_ = 6.4	− 1.8 [− 6.0 to 2.4] BF_10_ = 0.205
CDR 0 + 0.5 cases		
Basal forebrain	59.8 [− 10.6 to 130.6] BF_10_ = 0.851	− 47.6 [− 106.2 to 11.8] BF_10_ = 0.457
Hippocampus	12.8 [− 2.5 to 5.8] BF_10_ = 0.797	− 5.8 [− 18.7 to 7.0] BF_10_ = 0.186
Thalamus	6.1 [1.4 to 10.7] BF_10_ = 6.3	− 2.5 [− 6.4 to 1.4] BF_10_ = 0.286

β—unstandardized parameter estimates for the association between volume and cognitive function from the ANCOVA model, controlling for age, sex, education, CDR score (in CRD 0 + 0.5 cases), and mutation carrier status.

### Voxel based analysis of MRI data

**Figure 4:**
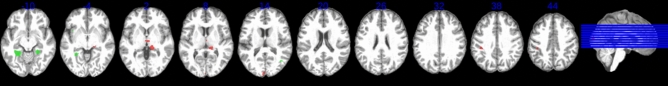
Association of carrier status with voxel-wide grey matter volume. Relative increase of signal in mutation carriers vs. non-carriers (green) and relative decrease in mutation carriers (red). Cluster of at least 50 voxels with p < 0.001. Blue figures on top of each scan indicate the z-coordinate in MNI space, corresponding to the level of the axial sections. Right of image is right of brain (view from superior).

We found small areas of reduced volumes in PSEN1 E280A carriers compared with non-carriers in right predominant medial thalamus, consistent with the region of interest based analyses (Fig. 4), as well as small areas of increased brain volumes in carriers vs. non-carriers in bilateral fusiform gyrus, middle temporal gyrus and cerebellum.

### Association of carrier status with regional metabolism

**Figure 5:**
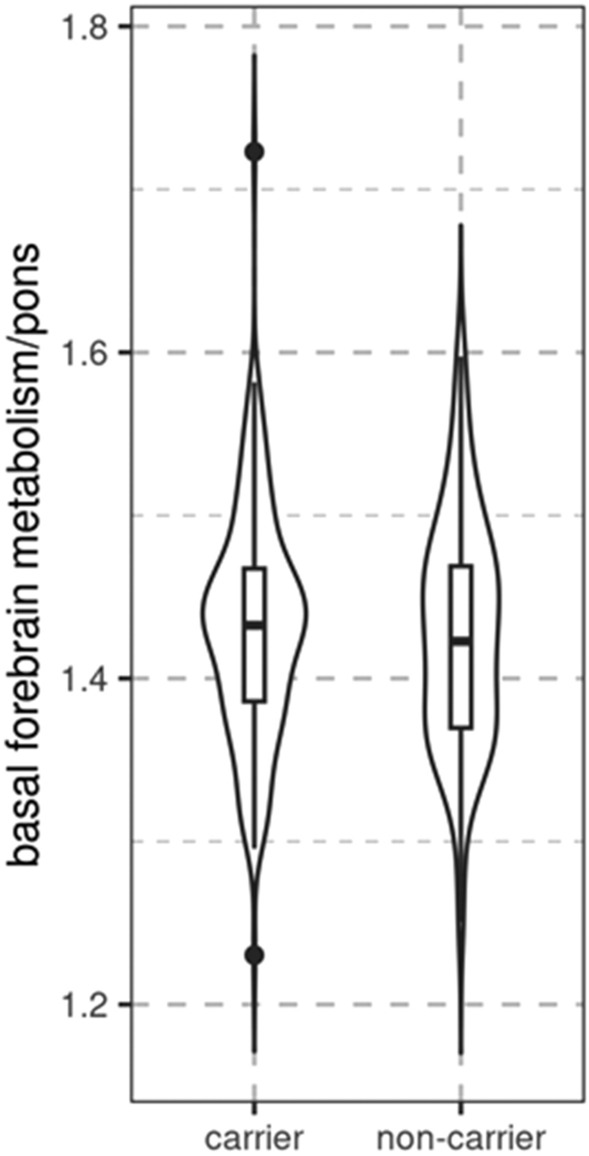
Association of carrier status with basal forebrain FDG-PET signal. Boxplot and violin plot featuring distribution of FDG-PET signal of the basal forebrain normalized to pons signal, according to mutation carrier status.

In an ANCOVA model, evidence was in favor of no difference between mutation carriers and non-carriers in pons normalized basal forebrain signal (BF_10_ = 0.167), controlling for age, sex, and CDR score, suggesting a relatively preserved basal forebrain metabolism in mutation carriers (Fig. 5). We found anecdotal evidence against a difference of hippocampus metabolism (BF_10_ = 0.582) and moderate evidence against a difference of thalamus metabolism (BF_10_ = 0.165) in mutation carriers compared with non-carriers.

### Voxel based analysis of FDG-PET data

**Figure 6:**
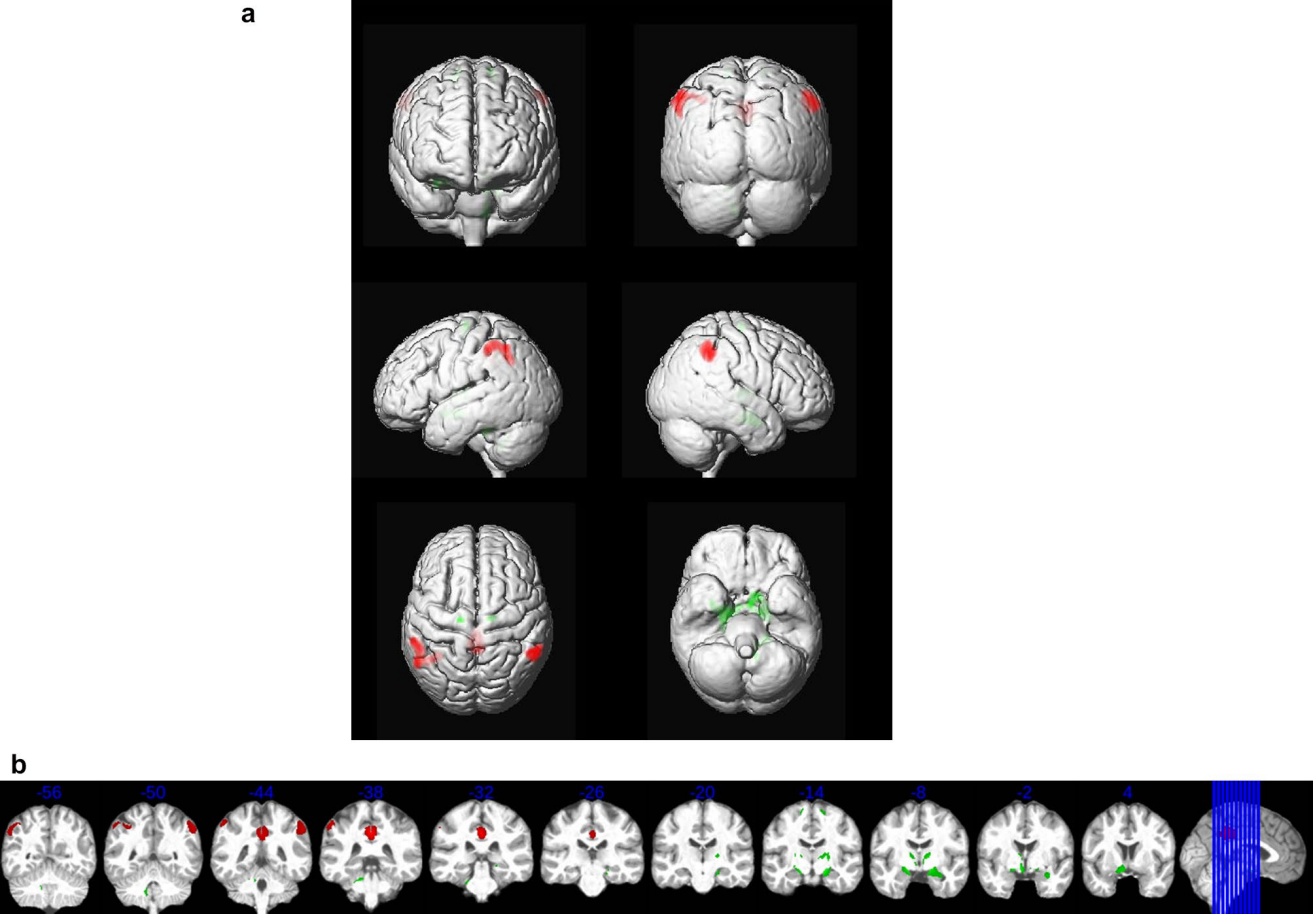
Voxel-wise association of carrier status with FDG-PET signal. (**a**) Effects projected on the rendered surface of an MRI scan in MNI space. Relative increase of signal in mutation carriers vs. non-carriers (green) and relative decrease in mutation carriers (red). Cluster of at least 50 voxels with p < 0.001. (**b**) Projection on coronal brain slices. Relative increase of signal in mutation carriers vs. non-carriers (green) and relative decrease in mutation carriers (red). Cluster of at least 50 voxels with p < 0.001. Blue figures on top of each coronal scan indicate the y-coordinate in MNI space, corresponding to the level of the coronal sections. Right of image is right of brain (view from posterior).

We found reduced globally-normalized metabolism in bilateral superior parietal and posterior cingulate cortex, and increased metabolism in cerebellum and basal forebrain regions in mutation carriers (Fig. 6).

### Amyloid sensitive AV45-PET data across groups

K-means clustering with R command kmeans resulted in two clusters with a threshold of approximately > 1.12 for amyloid positivity (Supplementary Fig. 1). We found extreme evidence in favor of an effect of carrier status on amyloid positivity (contingency table test, BF_10_ = 2.2 × 10^19^) with 57% of the mutation carriers being amyloid positive but none of the non-mutation carriers, see Supplementary Table 1 for details.

### Association of volumes with global AV45-PET signal

We found inconclusive evidence for an association of global AV45-PET signal with normalized basal forebrain (BF_10_ = 2.1) or hippocampus volume (BF_10_ = 1.4), but very strong evidence in favor of an association of global AV45-PET signal with thalamus volume (BF_10_ = 99.0). More amyloid signal was associated with smaller thalamus volume, after controlling for mutation carrier status. Details can be found in Supplementary Fig. 2. The effect of mutation carrier status on thalamus volume was fully mediated by the global AV45-PET signal, accounting for 87% of the covariance (Supplementary Fig. 3).

## Discussion

Contrary to our primary hypothesis, we found evidence against an association of mutation carrier status with basal forebrain volume. In sporadic AD, atrophy of cholinergic basal forebrain is an early event and detectable in prodromal and even in preclinical stages of the disease^5–9^. Here, we found evidence against atrophy of the basal forebrain in preclinical and prodromal ADAD related to a PSEN1 E280A mutation. Basal forebrain atrophy has not been studied before in ADAD and also pathological studies have not investigated involvement of cholinergic basal forebrain neurons in ADAD cases. A cortical nicotinergic receptor deficit has been described in Swedish mutation carriers, however, this was based on autopsy in advanced stages of AD^31^. Preclinical studies demonstrated pathology of the cholinergic basal forebrain in transgenic mice with double or single amyloid precursor protein (APP) and presenilin1 mutations^32–35^. Specifically, in addition to confirming the presence of amyloid plaques, these studies showed a significant decrease in hippocampal choline acetyltransferase (ChAT) activity^32^, metabolic changes and DNA damage in basal forebrain^35^, no evidence of tau pathology but extensive inflammatory glial responses and increased trophic effects^34^. In contrast, one study analyzing ChAT activity, ChAT mRNA level, cholinergic neuron number and receptor binding in mutant PSEN1, APP and PSEN1/APP mice showed evidence of intact basal cholinergic innervation, even in the presence of extensive amyloid pathology^36^. Our results raise the question whether these findings are transferable to humans with such mutations.

Similar to the basal forebrain, we found evidence against atrophy of the hippocampus in the PSEN1 E280A mutation carriers. This finding in the relatively large cohort agrees with previous studies on subsamples of less than 30 asymptomatic carriers and 30 noncarriers each of the Colombian kindred that revealed no statistically significant reductions in hippocampal volume^19,20,24^. Likewise, a study in ADAD mutations other than PSEN1 E280A found no hippocampus atrophy in nine asymptomatic mutation carriers compared with nine control subjects, and even when 12 MCI mutation carriers were considered, the hippocampus was spared atrophy^21^. In carriers of different APP, PSEN1, and PSEN2 mutations, hippocampal atrophy was found from approximately 5 years before^17^ to 8 years after the expected onset of the disease^37^, indicating a large variation of hippocampus atrophy trajectories across different mutations.

Interestingly, children and adolescents with the Colombian PSEN1 E280A mutation at age 9–17 years showed increased hippocampus, parahippocampus, and parietal and temporal lobe volumes compared with non-carriers^38^. Similarly, a study of individuals with different PSEN1 mutations found a significant increase of cortical thickness in parietotemporal regions in six asymptomatic mutation carriers on average 9.9 years before expected age of onset^39^. This is also consistent with a study in seven PSEN1 mutation carriers, distinct from the Colombian kindred, who showed accelerated hippocampus atrophy with transition to a symptomatic stage, but did not differ from controls in hippocampus volumes before this transition^18^. Similarly, in a cross-sectional analysis, cortical thickness in PSEN1 E280A carriers was higher in children and adolescent mutation carriers compared to age-matched noncarriers^40^. The underlying mechanisms of the volume increases in young asymptomatic individuals with PSEN1 mutations are currently unresolved. The PSEN1 E280A mutation may be associated with early developmental changes or neuroinflammation with glial activation or neuronal hypertrophy^41^ in response to neurotoxic amyloid and lead to early apparent increase of volume in AD vulnerable regions, only later followed by neurodegeneration and related brain atrophy. This also fits with the observation from mutation carriers of the DIAN cohort that did not show differences to non-carriers in hippocampus volume at an asymptomatic baseline, but mild rates of hippocampus atrophy were followed by a strong increase of rates of volume loss only after symptom onset^42,43^. The lack of hippocampal atrophy in our analysis may therefore reflect the asymptomatic to early symptomatic stage of PSEN1 E280A mutation carriers, in which there may be a transition from a developmental increase to a neurodegenerative decrease in hippocampal volume.

A possible effect similar to that seen in the hippocampus, with increased volume in young asymptomatic ADAD mutation carriers, has not yet been investigated for the basal forebrain, leaving open whether the lack of atrophy in our analysis represents a possible transitional phenomenon from developmental changes to neurodegeneration.

Several studies, mostly using voxel-based analyses, reported atrophy of the thalamus in asymptomatic mutation carriers^21–23^, including individuals of the Colombian kindred^24^, reviewed in^44^. Early atrophy of the thalamus in ADAD is consistent with the early deposition of amyloid in this region and the striatum as shown using amyloid sensitive PIB-PET imaging in mutation carriers from the DIAN study^22^. Here, we found that atrophy of the thalamus, but not the basal forebrain or hippocampus^20^, was associated with a decrease in delayed memory performance in mutation carriers, both when considering all cases and when considering only asymptomatic cases. One study in a small sample of the Colombian kindred did not find a difference in whole thalamus volume and thalamic subregion volumes between mutation carriers and non-carriers, despite numerically larger volumes in the noncarriers^45^.

Consistent with our primary hypothesis, we found evidence for a preserved metabolism of basal forebrain in mutation carriers, consistent with previous studies in sporadic prodromal AD^13,14^. Regions with relatively preserved metabolism included basal forebrain, thalamus and hippocampus in the voxel based analysis. In contrast, other PSEN1 mutations showed pronounced hypometabolism of hippocampus in asymptomatic stages^46^, however, basal forebrain metabolism had not been assessed before. Hypometabolism in our analysis was mainly detected in superior parietal cortex, precuneus, and posterior cingulate gyrus, resembling the topography of cortical thickness reductions in the DIAN cohort asymptomatic mutation carriers^47^ and the typical pattern of hypometabolism found in sporadic cases with amnestic or amyloid positive MCI^48^.

Amyloid load was much more pronounced in the mutation carriers than the non-carriers, consistent with the presence of a PSEN1 mutation and the young age of the cohort that implied a low risk for cerebral amyloidosis in non-mutation carriers. Different to previous analyses in sporadic prodromal and preclinical AD cases^10–12^, we only found anecdotal evidence for an association of global amyloid load with basal forebrain volume. In contrast, evidence was very strong for an association of global amyloid load with thalamus volume, and amyloid load fully mediated the association of mutation carrier status with thalamus volume. This is consistent with the pathogenic role of the PSEN1 E280A mutation leading to early-onset cerebral amyloidosis as the main determinant of subsequent neurodegeneration and cognitive decline^49^.

### Limitations

We note the following limitations of this study: we lacked longitudinal follow-up since the study participants were included in the ongoing API ADAD Colombia trial so that access to longitudinal data was not possible. We also lacked information on the expected age of onset for the asymptomatic mutation carriers, which would have been very interesting to study in association with brain volumes and metabolism. The homogeneity of the genetic background is both an advantage and a disadvantage. It shows that the pathomechanistic effects of the PSEN1 E280A mutation lead to early involvement of thalamus but not of basal forebrain or hippocampus. It needs, however, to be shown if these results generalize to other presenilin1 mutations and even to presenilin2 and APP mutations. Basal forebrain volume and metabolism are surrogate markers for the integrity of the cholinergic basal forebrain, but not a direct estimate of neuronal structural and functional integrity^3^. However, the methods applied here match similar studies in sporadic AD allowing a direct comparison.

## Conclusions

In this study, basal forebrain volume was not decreased in non-demented mutation carriers for ADAD and basal forebrain metabolism was relatively preserved. Early changes in delayed memory were associated with thalamus but not basal forebrain and hippocampus volume pointing to a different involvement of subcortical brain regions and relative sparing of cholinergic projection sites in prodromal ADAD compared with sporadic AD. This study highlights the importance of alternative disease mechanisms in ADAD and sporadic AD, at least for the PSEN1 E280A mutation, that may be relevant to understand different pathogenic roles of amyloid pathology for regional brain atrophy and metabolic changes in early stages of ADAD and sporadic AD. These findings may also have implications for considering initiation of cholinergic treatment not only in prodromal sporadic AD, but also in prodromal cases of ADAD.

## Material and methods

### Data source

Data were made available from the Alzheimer’s Prevention Initiative (API) Autosomal-Dominant Alzheimer’s Disease Colombia Trial (NCT01998841) baseline data^50^. The trial design has been described before^51^. Originally, 252 participants had been enrolled, however, data on 10 participants had to be excluded already from the trial baseline data presentation^52^ to protect their confidentiality, genetic status, and trial integrity. Informed consent was obtained from all participants and study partners before any study related procedures were conducted. The trial was approved by the Colombian Health Authority (Instituto Nacional de Vigilancia de Medicamento). All consent procedures were conducted in accordance with international and local ethics committee standards and after ethics committee approval. All research was performed in accordance with relevant guidelines and regulations, including the Helsinki Declaration of 1975 and its later amendments.

Adherence to STROBE reporting guidelines is documented in Supplementary Table 2.

### Participants

The API Colombia trial included individuals who carry the PSEN1 E280A autosomal-dominant mutation^15^, do not meet criteria for MCI^53^ or dementia due to AD^54^, and are between ≥ 30 years and ≤ 60 years of age^51^. In addition, age- and sex matched members of the same kindred without the mutation were included. Detailed inclusion and exclusion criteria can be found in^51^.

### Cognitive measures

Measures included Clinical Dementia Rating (CDR) global score^55^ which we used for stratifying participants in unimpaired (CDR = 0) and slightly impaired (CDR = 0.5) samples. In addition, we used the Repeatable Battery for the Assessment of Neuropsychological Status (RBANS) Memory and attention scores^56^.

### Image data acquisition

#### MRI data acquisition

Volumetric MR imaging data were acquired on a 1.5-T imaging system (Avanto; Siemens) with a T1-weighted, magnetization-prepared, rapid-acquisition, gradient-echo pulse sequence (echo time, minimum full; flip angle, 8°; number of excitations, 1; field of view, 22 cm; imaging matrix, 192 × 192 pixels; and section thickness, 1.2 mm).

#### FDG PET data acquisition

FDG-PET images were acquired on a Siemens/CTI Biograph PET/CT system, using intravenous administration of 5 mCi (185 million Bq) of FDG after a 30-min radiotracer uptake period when resting with open eyes in a darkened room, followed by a 30-min dynamic emission scan (six 5-min frames). Images were reconstructed with computed tomographic attenuation correction.

#### Amyloid PET data acquisition

Florbetapir scans were acquired on the same Siemens Biograph PET/CT system as the FDG-PET data, using an intravenous bolus injection of ~ 11 mCi (9.3–14.7 mCi) of florbetapir, a CT scan for correction of radiation attenuation, a 50-min radiotracer uptake-period, and a 20-min dynamic emission scan in four frames (4 × 300 s)^57^. PET images were reconstructed using an ordered subset expectation maximization (OSEM) algorithm and attenuation-corrected, frames were evaluated for adequate count statistics and absence of head motion^57^.

### Image data processing

#### MRI data

MRI data were processed by using statistical parametric mapping (SPM12, Wellcome Trust Center for Neuroimaging) and the CAT12.3-toolbox (http://dbm.neuro.uni-jena.de/cat) implemented in MATLAB R2019 (MathWorks, Natick, MA). First, MRI scans were automatically segmented into grey matter (GM), white matter (WM) and cerebrospinal fluid (CSF) partitions of 1.5 mm isotropic voxel-size using the prior free Adaptive Maximum A Posterior (AMAP) segmentation routine of the CAT12-toolbox. The resulting GM and WM partitions of each subject in native space were then high-dimensionally registered to the MNI reference template using the DARTEL algorithm^58^. Individual flow-fields resulting from the DARTEL registration to the reference template were used to warp the GM segments and voxel-values were modulated for volumetric changes introduced by the high-dimensional normalization, such that the total amount of GM volume present before warping was preserved. For extraction of basal forebrain volume, we used a cytoarchitectonic map of BF cholinergic nuclei in MNI space, derived from combined histology and MRI of a post-mortem brain, as described previously^59^. Hippocampus and thalamus volumes were derived using the Hammers brain atlas regions of interest^60^.

#### PET data

Images were preprocessed using SPM12 implemented in Matlab 2019^61^. First, each subject’s averaged PET frames were co-registered to their corresponding T1-weighted MRI scan. Then, the coregistered PET images were spatially normalized to the MNI reference template using the deformation parameters derived from the normalization of their corresponding MRI scans.

For extraction of Florbetapir SUVR we used the Centiloid cortical mask and normalized the PET signal to the whole cerebellum Centiloid mask^62^. For FDG-PET we extracted regional SUVR values using the basal forebrain region and Hammers brain atlas regions of interest for hippocampus and thalamus^60^, and normalized the PET signal to the signal of the pons.

### Statistical analysis

For region of interest based analyses we used Bayesian ANCOVA with *Bayes factor (BF) hypothesis testing* with volume or metabolism as dependent variable, mutation carrier status as independent variable, and age, sex, CDR score, and (for analyses of cognitive scores) education as confounders, to compare the alternative hypothesis against the null hypothesis (i.e., the assumption that there is an effect of carrier status, H_1_)^26,27^, as implemented in *Jeffreys*’ *Amazing Statistics Program* (JASP Version 0.16.4), available at jasp-stats.org.

We report the Bayes Factor (BF_10_) quantifying evidence in favor of the alternative hypotheses. Three conclusions are possible within the Bayesian framework^26^: support for either the null hypothesis (BF_10_ ≤ 0.33), support for the alternative hypothesis (BF_10_ > 3), or inconclusive evidence (BF_10_ between 0.33 and 3). We applied the following evidence categories: a BF_10_ above 3 provides “substantial evidence”, a BF_10_ above 10 provides “strong evidence”, a BF_10_ above 30 provides “very strong evidence” and a BF_10_ above 100 provides “extreme evidence” against the null model^63^.

For data driven analysis we conducted voxel based analysis of grey matter maps and normalized FDG-PET data scaled to pons signal, respectively, using spm12, implemented in Matlab version 2020a. We regressed voxel-wise grey matter or FDG signal on carrier status, controlled for age, sex, and CDR score. We considered clusters of at least 50 voxel passing an uncorrected p-value of 0.001.

### Supplementary Information

Supplementary Information.

## Data Availability

Data are available from the authors upon reasonable request and with the permission of the Banner Alzheimer's Foundation. The Alzheimer’s Prevention Initiative website hosts materials and details regarding the API ADAD Trial and data sharing process for review or download at https://alzheimerspreventioninitiative.com.
