# Supplementary material for: Basal forebrain volume and metabolism in carriers of the Colombian mutation for autosomal dominant Alzheimer’s disease
Source: Sci Rep. 2024 May 17;14:11268. doi: 10.1038/s41598-024-60799-9 (PMC11101449; doi:10.1038/s41598-024-60799-9)
Supplement: Supplementary file 1 — Supplementary Information. [file 41598_2024_60799_MOESM1_ESM.docx]

**Supplementary Material**

**Supplementary Table 1: Distribution of amyloid positivity across mutation carrier status**

| **PSEN1 E280A** | **AV45 negative** | **AV45 positive** | **Total** |
| --- | --- | --- | --- |
| carrier | 71 | 96 | 167 |
| noncarrier | 75 | 0 | 75 |
| Total | 146 | 96 | 242 |

**Supplementary Table 2: Checklist of items for cross-sectional studies according to STROBE guildelines^1^**

|  |  | Item No | Recommendation | Page No |
| --- | --- | --- | --- | --- |
| **Title and abstract** |  | 1 | (*a*) Indicate the study’s design with a commonly used term in the title or the abstract | 2 |
|  |  |  | (*b*) Provide in the abstract an informative and balanced summary of what was done and what was found | 2 |
|  | Introduction | | | |
| Background/rationale |  | 2 | Explain the scientific background and rationale for the investigation being reported | 3-4 |
| Objectives |  | 3 | State specific objectives, including any prespecified hypotheses | 3-4 |
|  | Methods | | | |
| Study design |  | 4 | Present key elements of study design early in the paper | 12 |
| Setting |  | 5 | Describe the setting, locations, and relevant dates, including periods of recruitment, exposure, follow-up, and data collection | 12 |
| Participants |  | 6 | (*a*) Give the eligibility criteria, and the sources and methods of selection of participants | 12 |
| Variables |  | 7 | Clearly define all outcomes, exposures, predictors, potential confounders, and effect modifiers. Give diagnostic criteria, if applicable | 12 - 13 |
| Data sources/ measurement |  | 8 | For each variable of interest, give sources of data and details of methods of assessment (measurement). Describe comparability of assessment methods if there is more than one group | 12 - 14 |
| Bias |  | 9 | Describe any efforts to address potential sources of bias | NA |
| Study size |  | 10 | Explain how the study size was arrived at | NA |
| Quantitative variables |  | 11 | Explain how quantitative variables were handled in the analyses. If applicable, describe which groupings were chosen and why | 14 - 15 |
| Statistical methods |  | 12 | (*a*) Describe all statistical methods, including those used to control for confounding | 14 - 15 |
|  |  |  | (*b*) Describe any methods used to examine subgroups and interactions | 14 - 15 |
|  |  |  | (*c*) Explain how missing data were addressed | NA |
|  |  |  | (*d*) If applicable, describe analytical methods taking account of sampling strategy | NA |
|  |  |  | (*e*) Describe any sensitivity analyses | 14 - 15 |
|  | Results | | | |
| Participants |  | 13 | (a) Report numbers of individuals at each stage of study—eg numbers potentially eligible, examined for eligibility, confirmed eligible, included in the study, completing follow-up, and analysed | 5 |
|  |  |  | (b) Give reasons for non-participation at each stage | 12 |
|  |  |  | (c) Consider use of a flow diagram | NA |
| Descriptive data |  | 14 | (a) Give characteristics of study participants (eg demographic, clinical, social) and information on exposures and potential confounders | 5, 12 |
|  |  |  | (b) Indicate number of participants with missing data for each variable of interest | NA |
| Outcome data |  | 15 | Report numbers of outcome events or summary measures | NA |
| Main results |  | 16 | (*a*) Give unadjusted estimates and, if applicable, confounder-adjusted estimates and their precision (eg, 95% confidence interval). Make clear which confounders were adjusted for and why they were included | 5 - 7 |
|  |  |  | (*b*) Report category boundaries when continuous variables were categorized | NA |
|  |  |  | (*c*) If relevant, consider translating estimates of relative risk into absolute risk for a meaningful time period | NA |
| Other analyses |  | 17 | Report other analyses done—eg analyses of subgroups and interactions, and sensitivity analyses | 14 - 15 |
|  | Discussion | | | |
| Key results |  | 18 | Summarise key results with reference to study objectives | 8 |
| Limitations |  | 19 | Discuss limitations of the study, taking into account sources of potential bias or imprecision. Discuss both direction and magnitude of any potential bias | 10 - 11 |
| Interpretation |  | 20 | Give a cautious overall interpretation of results considering objectives, limitations, multiplicity of analyses, results from similar studies, and other relevant evidence | 11 |
| Generalisability |  | 21 | Discuss the generalisability (external validity) of the study results | 10 - 11 |
|  | Other information | | | |
| Funding |  | 22 | Give the source of funding and the role of the funders for the present study and, if applicable, for the original study on which the present article is based | 16 |

NA – not applicable

**Supplementary figure legends**

**Supplementary Figure 1: Distribution of AV45-PET signal**

Histogram and superimposed frequency distribution function for the global amyloid PET signal, corresponding to a bivariate Gaussian distribution. The vertical red line indicates the threshold for positive and negative amyloid values, respectively, derived from k-means clustering.

**Supplementary Figure 2: Association of brain volumes with global amyloid signal**

Scatterplot of volumes of basal forebrain (upper row), hippocampus (middle row), c) thalamus (lower row) regressed on global amyloid PET SUVR according to mutation carrier status.

**Supplementary Figure 3: Mediation of mutation carrier effect on thalamus volume by global amyloid signal**

Mediation model for the direct effect of carrier status on thalamus volume and the indirect effect mediated by global amyloid SUVR. Numbers indicate the standardized regression coefficients and their 95% credibility intervals, asterisks indicate coefficients whose credibility interval excludes zero

**Supplementary Figure 1: Distribution of AV45-PET signal**


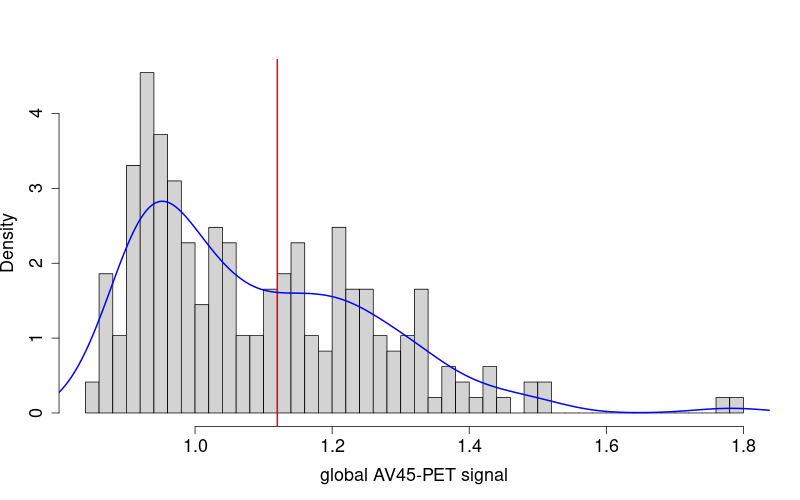


**Supplementary Figure 2: Association of brain volumes with global amyloid signal**


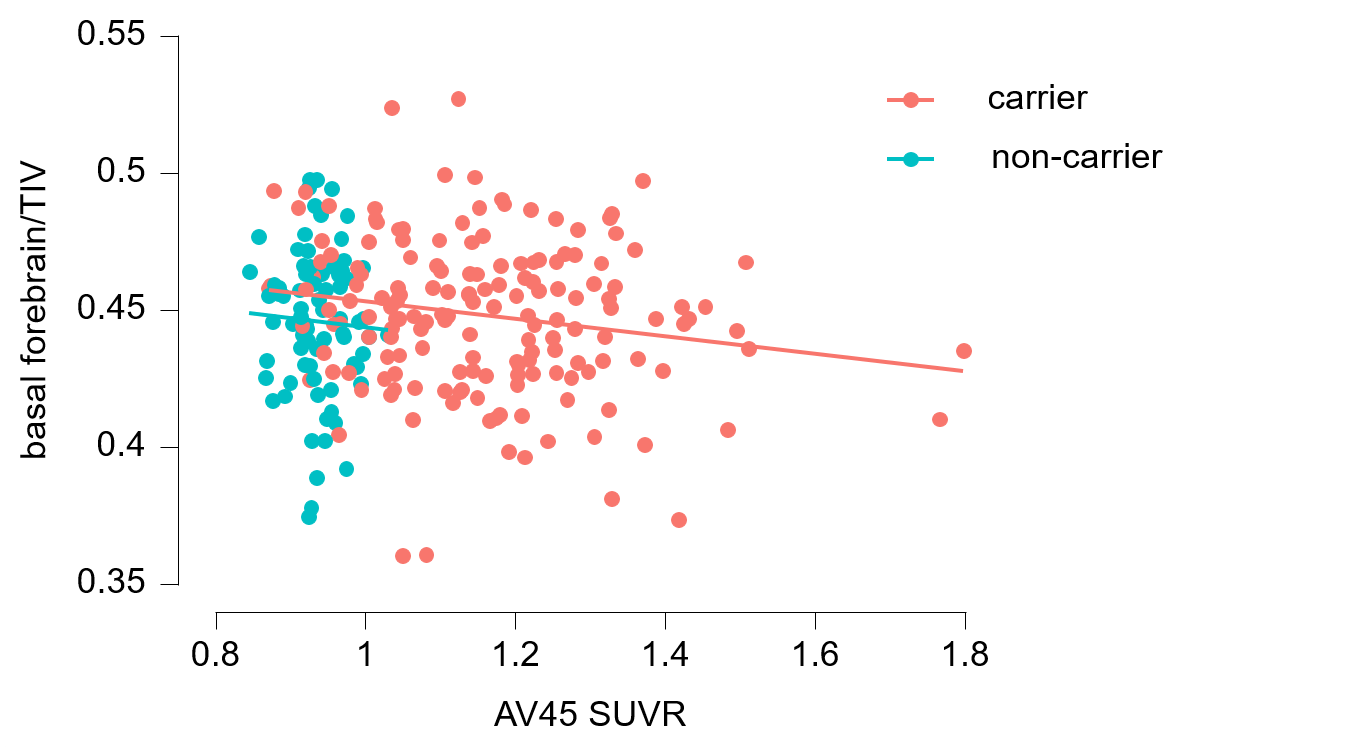


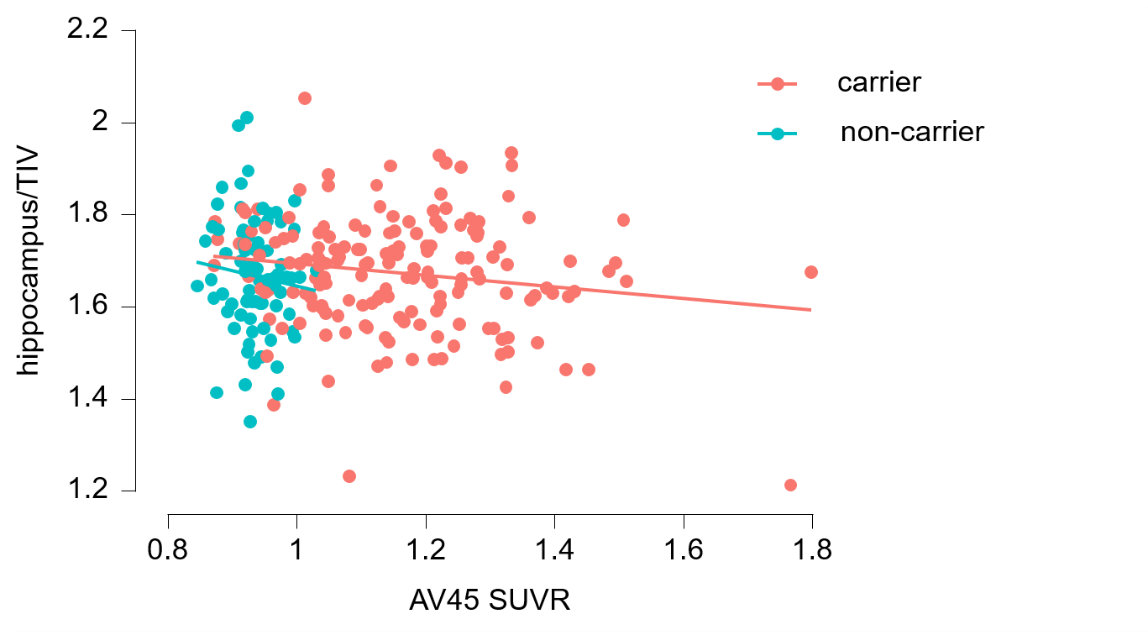


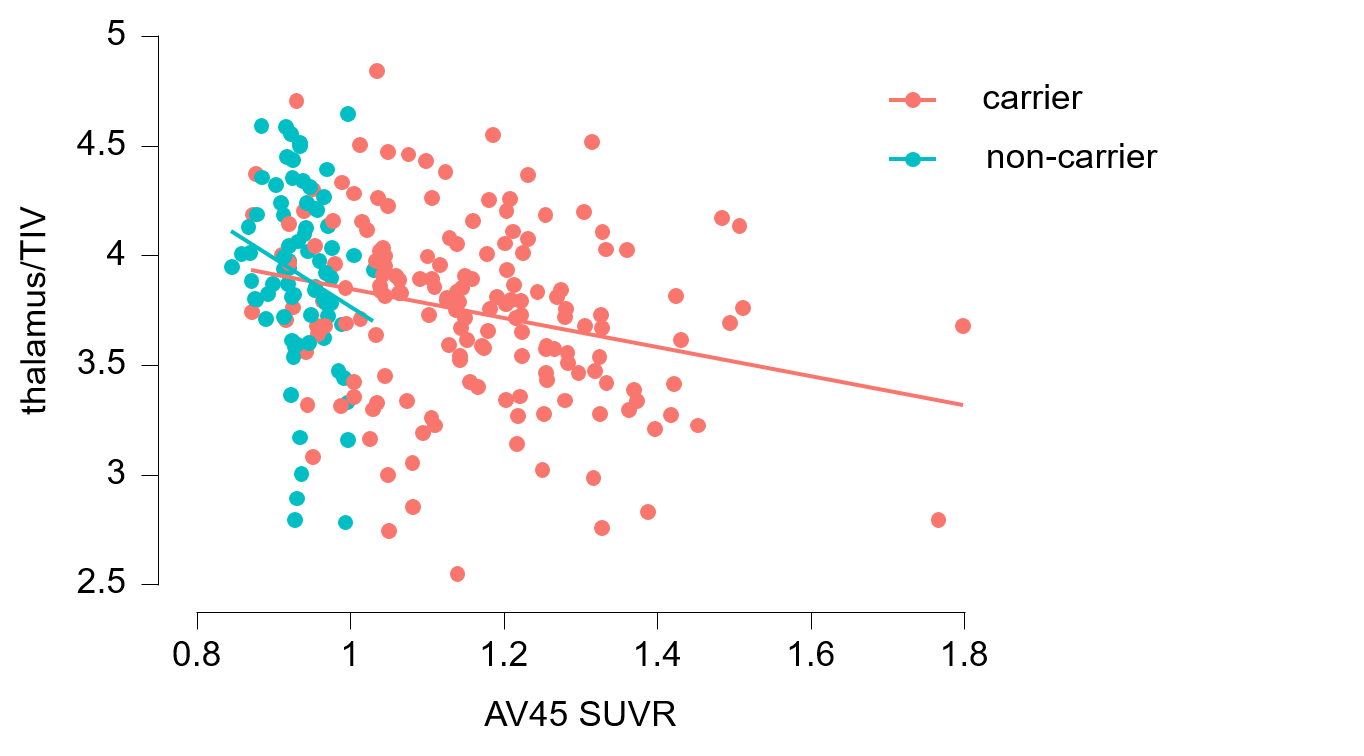


**Supplementary Figure 3: Mediation of mutation carrier effect on thalamus volume by global amyloid signal**

**
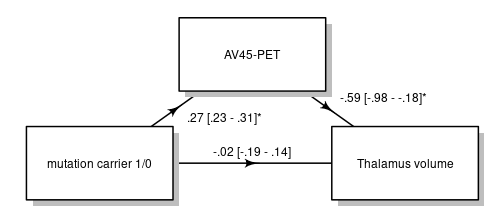
**

**References:**

1 von Elm, E. *et al.* The Strengthening the Reporting of Observational Studies in Epidemiology (STROBE) statement: guidelines for reporting observational studies. *Lancet* **370**, 1453-1457, doi:10.1016/S0140-6736(07)61602-X (2007).
